# On the way home: a BCI-FES hand therapy self-managed by sub-acute SCI participants and their caregivers: a usability study

**DOI:** 10.1186/s12984-021-00838-y

**Published:** 2021-02-25

**Authors:** Anna Zulauf-Czaja, Manaf K. H. Al-Taleb, Mariel Purcell, Nina Petric-Gray, Jennifer Cloughley, Aleksandra Vuckovic

**Affiliations:** 1 Biomedical Engineering Research Division, University of Glasgow, Glasgow, UK; 2 Wasit University, Wasit, Iraq; 3 Queen Elizabeth National Spinal Injuries Unit, Elizabeth University Hospital, Glasgow, Queen UK

**Keywords:** Electroencephalography, Spinal cord injury, Brain computer interface, Functional electrical stimulation, Rehabilitation, Usability

## Abstract

**Background:**

Regaining hand function is the top priority for people with tetraplegia, however access to specialised therapy outwith clinics is limited. Here we present a system for hand therapy based on brain-computer interface (BCI) which uses a consumer grade electroencephalography (EEG) device combined with functional electrical stimulation (FES), and evaluate its usability among occupational therapists (OTs) and people with spinal cord injury (SCI) and their family members.

**Methods:**

*Users*: Eight people with sub-acute SCI (6 M, 2F, age 55.4 ± 15.6) and their caregivers (3 M, 5F, age 45.3 ± 14.3); four OTs (4F, age 42.3 ± 9.8). *User Activity*: Researchers trained OTs; OTs subsequently taught caregivers to set up the system for the people with SCI to perform hand therapy. Hand therapy consisted of attempted movement (AM) of one hand to lower the power of EEG sensory-motor rhythm in the 8-12 Hz band and thereby activate FES which induced wrist flexion and extension. *Technology*: Consumer grade wearable EEG, multichannel FES, custom made BCI application. *Location*: Research space within hospital. *Evaluation*: donning times, BCI accuracy, BCI and FES parameter repeatability, questionnaires, focus groups and interviews.

**Results:**

*Effectiveness*: The BCI accuracy was 70–90%. *Efficiency*: Median donning times decreased from 40.5 min for initial session to 27 min during last training session (N = 7), dropping to 14 min on the last self-managed session (N = 3). BCI and FES parameters were stable from session to session. *Satisfaction*: Mean satisfaction with the system among SCI users and caregivers was 3.68 ± 0.81 (max 5) as measured by QUEST questionnaire. Main facilitators for implementing BCI-FES technology were “seeing hand moving”, “doing something useful for the loved ones”, good level of computer literacy (people with SCI and caregivers), “active engagement in therapy” (OT), while main barriers were technical complexity of setup (all groups) and “lack of clinical evidence” (OT).

**Conclusion:**

BCI-FES has potential to be used as at home hand therapy by people with SCI or stroke, provided it is easy to use and support is provided. Transfer of knowledge of operating BCI is possible from researchers to therapists to users and caregivers.

*Trial registration* Registered with NHS GG&C on December 6th 2017; clinicaltrials.gov reference number NCT03257982, url: https://clinicaltrials.gov/ct2/show/NCT03257982.

## Introduction

In recent years, there has been a shift towards healthcare at home away from hospital, it is an appealing strategy reducing healthcare system costs [1]. Among the many who could potentially benefit from such innovations are persons suffering from traumatic spinal cord injury (SCI). A significant portion of these people are young and live with the condition for decades [2]. Depending on the neurological level and severity of their injury they may be significantly or totally dependant on carers for activities of daily living (ADL). For such persons achieving maximum hand function is crucial for ADL, and studies have shown hand function is the top priority for people with tetraplegia [3]. While the greatest degree and most rapid recovery of function has been shown to occur within the first year of injury [4], in a cost-conserving strategy, rehabilitation facilities discharge patients often within a few months of injury. This may limit the person’s recovery as access to physiotherapy and rehabilitation is limited outwith specialised centres and barriers to such treatment include the lack of social support, inadequate therapist knowledge about needs of people after SCI, and cost concerns [5]. Therefore accessible, easy to use, and effective solutions are needed to facilitate community rehabilitation for people with SCI.

Brain computer interface (BCI) technology has been combined with Functional Electrical Stimulation (FES) for rehabilitation after stroke and SCI, and has been shown to be effective in a clinical setting with some studies reporting significant motor improvements [6–10], while others neurological changes [11, 12]. Neuroplasticity via motor priming has been suggested as the underlying principle, meaning BCI facilitated motor cortex activation occurs for some time prior to FES induced motor response and sensory stimulus being delivered [13].

These BCI systems were designed to be administered by researchers in a clinical setting, however while people are in a hospital during the acute and sub-acute stage numerous obstacles such as limited time and the fragile physical and mental state of the users prevent effective BCI therapy [14]. These BCI systems also cannot be easily transferred to users as a community healthcare tool as they typically require expert knowledge to operate. Hence over the last few years studies have emerged which have paved the way for introducing BCI technology into the end users’ homes. The focus has shifted from BCI performance to ease of use and feasibility of the transfer of knowledge of such technology to the users and their immediate caregivers.

In light of this, caregiver opinions of particular BCI systems for communication have been assessed in addition to those of end users [15, 16]. For users the effectiveness and accuracy of the system was important, while caregivers stressed the need for simplicity of the hardware setup and user friendliness of the user interface. Studies in which caregivers were actually taught and used a BCI communication system with the end user in a home setting did not report caregivers’ views of the technology [15, 17]. A BCI system called BackHome used for communication and other functions, was tested in a clinical setting by users, caregivers, and therapist, and was generally well received by all groups [18]. Other groups have combined BCI with Functional Electrical Stimulation (FES) as an assistive device. Most notably the MoreGrasp project debuted a BCI-FES neuroprosthesis worn on the arm by people with chronic SCI as an assistive device at home, rather than a rehabilitative device [19].

Other studies have involved caregivers and therapists via focus groups or questionnaires not necessarily using any particular BCI system but investigating general attitudes and barriers toward adopting BCI technology [20]–[22]. It has been shown that clinicians tend to be more cautious and critical of BCIs than the potential end users [21, 23] hence the concept of user centred design (UCD) has been applied BCIs and rehabilitative devices [18]. Since many assistive devices are abandoned completely by the users even though they perform the intended function [24, 25], such an approach to designing a BCI is essential in order for the technology to be used and effective. Hence to test the feasibility of introducing a BCI system as a community healthcare tool, information is needed about the needs and obstacles each of the three interest groups, therapists, caregivers, and end users experience during the process of learning and using the system in order to include them in the process of UCD.

Therefore in light of the above, the aim of the present study is to test the feasibility of transfer of knowledge of using a custom designed BCI-FES application based on consumer grade EEG technology.

We hypothesize that with adequate support and training, healthcare professionals will not only be able to use the system but will also be able to successfully train lay users (people with SCI and caregivers) to self-manage the therapy. Listed below are the objectives.

1. To present a custom made BCI-FES solution based on a consumer grade EEG device. We do so in *Methods* using a framework for reporting on BCI technology [35].
2. To evaluate the usability of the system among therapists, people after SCI and their caregivers in terms of efficiency, effectiveness, and satisfaction.
3. To investigate via interviews and focus groups the potential barriers and facilitators for adopting a self-administered BCI technology for neurorehabilitation at home.

## Methods

**Fig. 1:**
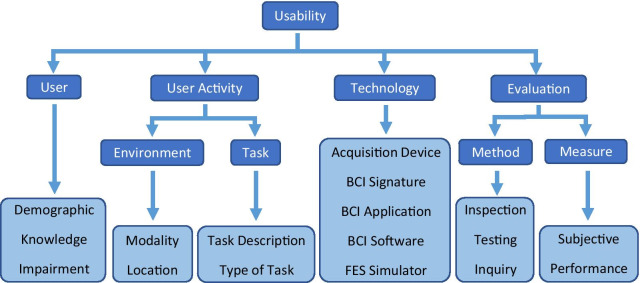
Usability framework as proposed by Rhiu et al. [26]. Two categories have been added, BCI Software and FES Stimulator, which are specific to the present study

Due to the volume and heterogeneity of emerging studies in BCI usability, Rhiu et al. [26] have composed a framework for reporting and categorising various aspects of the BCI technology, users, and data collection methods. This framework, with only minor modifications, allows us to describe our BCI-FES system for hand therapy custom designed to be accessible for the end users, as well as the methods employed. The BCI usability framework proposed by Rhiu et al. [26] is presented in Fig. 1, with the addition of categories “BCI Software” and “FES Stimulator” which are specific to this study.

The established constructs for evaluating usability in terms of efficiency, effectiveness, and satisfaction will be used to report results of both quantitative and qualitative measures of performance and user experience [27, 28].

### Users

The study involved three groups of users of BCI-FES: OTs, people with subacute SCI, and their caregivers (usually a family member). All participants were novice BCI users with no prior experience with any type of BCI or neurofeedback systems.

Overall eight people with SCI with their caregivers were recruited, though one pair withdrew after only one BCI-FES session, leaving limited data. Of the eight participants with SCI two were female, six male, mean age was 55.4 ± 15.6 years (min 20, max 73). Four were educated to secondary education level, four to post-secondary. Impairment characteristics were as follows: two C2 level injuries, four C4, two C5; four AIS C meaning sensory and motor incomplete injury with motor grade lower than 3 below the level of injury and four AIS D incomplete injury but motor grade greater than 3 below the level of injury [29]; and mean time after injury at recruitment was 12.4 ± 6.2 weeks (min 6, max 26). The median Manual Muscle Test (MMT) [30] scores across for flexor carpi radialis, extensor carpi radialis longus, flexor digitorum profundus, and extensor digitorum communis for the hand to which the BCI-FES was applied were 4− (min 0, max 4 +), 4 + (min 1 + , max 5), 3 + (min 0, max 4 +), and 2− (min 0, max 4−) respectively. The corresponding scores for the hand which was not trained were 4 (min 2−, max 4 +), 4 (min 2−, max 5), 4− (min 0, max 4), and 2− (min 0, max 4) respectively.

Of the caregivers recruited five were female and three male, mean age was 45.3 ± 14.3 years (min 25, max 70). Four had a secondary education and four post-secondary. The mean distance travelled from home to the hospital where the study took place was 37.5 ± 24.1 km (min 3.2, max 64.4).

A total of four OTs were recruited, all female, with a mean age of 42.3 ± 9.8 years (min 28, max 55). The mean number of years of experience as OT was 18.3 ± 10.0 (min 6, max 33) and had between 3 and 15 years of experience in administering FES in conventional hand therapy.

Ethical approval was granted by the National Health Service Greater Glasgow and Clyde Research Ethics Committee; clinical trial reference NCT03257982. All participants, people with SCI, caregivers and OTs, provided signed informed consent.

### User activity

**Fig. 2:**
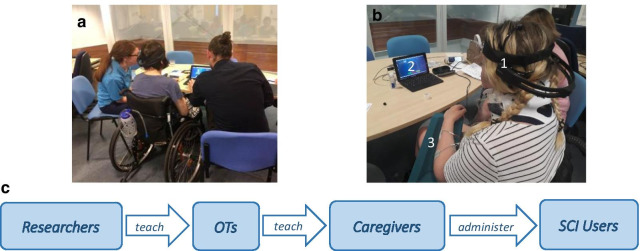
Users’ roles during BCI-FES sessions. User with caregiver and OT during BCI-FES a session in (**a**) and user with (1) EEG headset, (2) BCI program GUI, and (3) FES electrodes on arm in (**b**). In (**c**) schematic diagram of each user groups’ roles: OTs were first trained to use the BCI system by researchers, and then proceeded to teach caregivers how to set up the system for SCI users to perform the BCI-FES therapy

Each user group’s role during the study can be seen in Fig. 2. Researchers recruited OTs and taught them to use the system, then subsequently supervised and assessed each of the participants. OTs learned to use the BCI system in up to five hourly sessions and went on to teach each caregiver to operate it in up to five hourly training sessions also. Following successful training, the SCI user and caregiver pair had the option to complete up to 10 additional sessions independently (supervised by researcher but without OT) or to discontinue the study. If they chose to continue, the caregiver’s role was to set up the system for the person with SCI. SCI user’s role was to actually perform the BCI facilitated hand therapy.

### Task

**Fig. 3:**
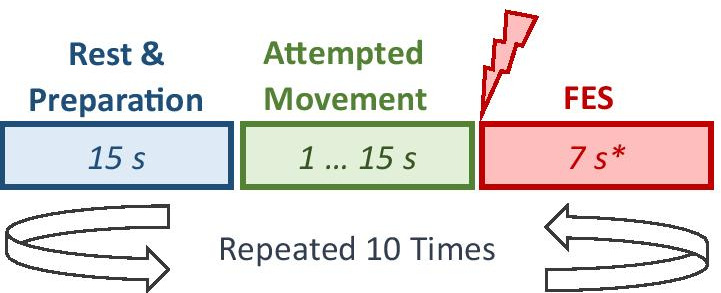
Schematic diagram of the course of one BCI-FES trial. (*) FES duration was typically 7–8 s, though this varied between users

The user was asked to move a pointer on a gauge displayed on screen towards a lower value, via attempted movement of their right or left hand, making this “a closed copy movement control task” [31]. The pointer represented real time alpha band (8–12 Hz) power as measured from the motor cortex of the corresponding hand, and FES was controlled by threshold time switch, i.e. when alpha power remained below a predefined threshold value for a set period of time (in this case 1 s). The lower power level corresponds to event related desynchornisation (ERD) which occurs in the motor cortex during executed or attempted movement. One BCI run was subdivided into 10 trials. Within one trial, after a rest period, the user was cued to attempt movement of their hand. They had minimum of 1 s and maximum 15 s to accomplish the task (Fig. 3). These values were heuristically determined based on our previous studies [31]. Users’ number of trials was limited 30 trials per hand per session, to avoid muscle fatigue.

### Environment and modality

In the present study, the location was a dedicated research space within a hospital for both the BCI training sessions and the second self-managed part by SCI users and caregivers. However intended environment for self-managed BCI is home.

Modality of stimulus was qualified as “multimodal”. Two cue modalities were utilised: the SCI users were provided with a visual execution cue, a tick which appeared on screen, and a simultaneous audio cue, high pitched ‘beep’ sound to signal the beginning of a trial.

The modality of feedback used was also multimodal. The visual scale on screen represented the real-time alpha band power while FES provided sensory feedback.

### Technology

Hardware consisted of a BCI and FES device. A proprietary user application for was developed in C++ to enable BCI control and communication between the EEG device and FES stimulator [32].

To acquire EEG signal during therapy sessions, the Emotiv EPOC (Emotiv Inc., USA) was used with a 128 Hz sampling rate. From the 14 channels of the headset, only two bipolar channels were used for real time neurofeedback: either approximately FC3 or CP3 for right hand training or FC4 and CP4 for left hand training, according to the international 10–10 standard system [33]. Emotiv EPOC was not originally designed to cover these electrode locations, therefore the headset was tilted back to allow sensors to be placed in these locations as measured from the nasion and inion for each user. Reference electrodes were in locations of PO7 and PO8 approximately, and ground electrodes on mastoid processes. Impedance was kept under 10kΩ.

For the initial and final EEG assessments GTech g.USBamp BioAmplifier (Guger Technologies, Austria) was used with sampling rate of 256 Hz and 16 EEG channels in the following locations: AFz, F3, Fz, F4, FC3, FCz, FC4, C3, Cz, C4, CP3, CPz, CP4, P3, Pz, P4 with ground on A1 and reference on A2 (L and R earlobes respectively). The impedance was kept under 5 kΩ.

**Fig. 4:**
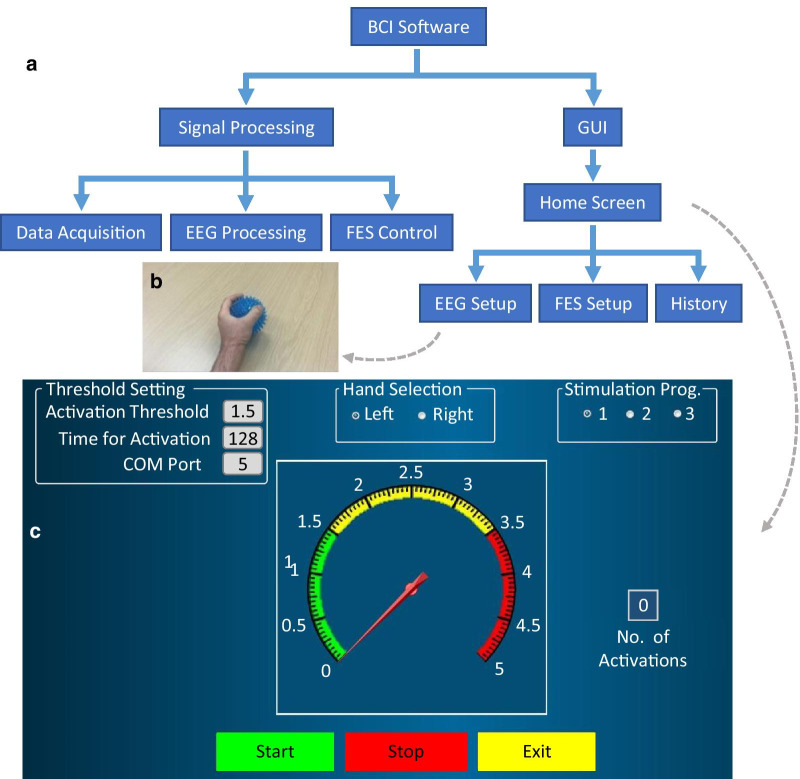
Aspects of the BCI-FES software. **a** Diagram of BCI software architecture and GUI aspects. **b** Screenshot of the training video. The hand squeezes the ball and relaxes. Repeated 10 times. **c** Main screen of the BCI programme. Setting options are in the upper portion of the window. The gauge is used for neurofeedback, ‘Start’ runs the programme, ‘Stop’ stops it and ‘Exit’ exits the BCI application

Figure 4a shows a diagram of the bespoke BCI software solution [32]. The GUI consisted of a main window from which users could control BCI-FES and navigate to separate ‘EEG Setup’ and ‘FES Setup’ windows. From the main window users could also access a ‘Usage Diary’ to visualise past use.

*Main Window* provided the following settings (Fig. 4c):

- *Training Threshold—*a value of alpha band power the user must stay below for Time Threshold (see below) in order to receive FES. The threshold value could be obtained using the Training video (described in EEG Setup Window below) or a value from a previous session could be reused.
- *Time Threshold—*time the alpha band power must be below the Training Threshold in order to activate FES
- *Hand Selection—*user selected left or right hand to be trained
- *Stimulation Program—*controlled the number of FES channels to be used. The user could choose up to four bipolar channels.

*EEG Setup Window* contained an automatic Training Threshold value suggestion, which was based on 10 repetitions of a 5 s “follow-along” video of a hand squeezing a ball (Fig. 4b). The suggested Training Threshold value was 1/3 of mean alpha band power during attempted movement, based on results of our previous study [31].

*FES Setup Window* was used to specify parameters for up to 4 independent FES bipolar channels including pulse width (typically 250 µs), stimulation amplitude (between 12 and 50 mA, set to achieve a visible muscle contraction), time between pulses, corresponding to the frequency of stimulation (33 Hz for all users), and duration of stimulation (typically between 3 and 5 s per channel) which was independent of EEG once the FES has been successfully triggered. In addition a start time for each of 4 channels could be set independently. In this way, a complex movement could be achieved, such as flexion and subsequent extension, separate flexion of wrist and hands, or separate movement of thumb and rest of the fingers [32].

The FES stimulator, Rehastim (Hasomed Ltd, Germany) allowed up to four channels to be used independently. Each user’s particular motor deficit was individually assessed by the OT. Most commonly the FES electrodes were placed on the forearm in order to activate the wrist and finger flexors and extensors in an alternating manner.

### Evaluation

Both testing and inquiry methods were utilised to assess the usability of the system through several subjective and performance (objective) measures. Each group of users was asked to provide subjective input regarding the BCI system in following ways.

- *Workload was measured by the NASA Task Load Index* [34] for SCI users, caregivers, and OTs. SCI users and caregivers were asked to complete a NASA TLX rating scale after each of their BCI sessions. OTs completed them after each of their training sessions (lead by researcher). The workload experienced gave a measure of the users’ progress during learning to use the system, indicating phases during which more support and improvements could be implemented.
- *Perceived usefulness of a device for home-based hand therapy questionnaire* was custom designed and given to SCI users and caregivers after their first BCI-FES session in order to capture their first impressions and expectations of the system. Perceived usefulness is a major factor in predicting intended use of a device, therefore this questionnaire measured perceived benefit to hand function and ease of use.
- *Quebec user evaluation of satisfaction with assistive technology (QUEST)* [35] questionnaire was completed by SCI users and caregivers after last training session with OT but before any additional sessions (after 4th or 5th session total). Satisfaction scores for specific aspects of the hardware and support given indicate areas for improvement, while users’ ratings of the device features reveal their priorities.
- *Focus group interviews* were conducted with OTs before recruitment of any users and caregivers, and after completion of all sessions with user pairs. These gave OTs an opportunity for feedback and critical discussion about their experience and future directions of the system.
- *Interviews* were carried out with SCI users and caregivers two or three times depending on whether or not they completed the optional 10 sessions after training. The interviews aimed to provide feedback regarding the system and gauge feasibility and interest in further development and use of the system at home. The first interview occurred after their first BCI-FES session. This interview assessed their general attitude towards technology and how they would look for information regarding new rehabilitation technology specifically. During the second interview, after completed training with OT, users and caregivers were asked about their understanding of how the system works, user friendliness, and their experience using the system. The final interview was conducted after the completion of the additional 10 sessions and focused again on the users’ and caregivers’ experience using the system, any user friendliness issues, and perceived benefit of extended therapy. During analysis interviews were printed verbatim and inspected by two researchers independently to identify the main topics. Researchers then agreed on the main topics, which are presented in the results section.

Several performance (objective) measures aimed to assess the efficiency and effectiveness of the BCI system.

- *Multichannel EEG assessment* was performed prior to starting the BCI-FES training and therapy. Initial and final resting state baseline recordings were performed. During these users had their eyes open while looking at a fixation cross on screen for two minutes, and two minutes with eyes closed. This was repeated twice at the start and twice at the end of the assessment. During the assessment each SCI user was asked to preform attempted movement of each of their hands. A cue based paradigm was used [36] and a total of 60 trials of each hand were performed, split up into 5 subsections each containing 12 trials per hand. One trial lasted a total of 6 s: −3 < t < −1 s a blank screen, a warning cue for −1 s < t < 3 s in the form of a cross in the middle of the screen, and an execution cue for 0 < t < 2 s in a form of an arrow pointing left or right, corresponding to the left and right hand movement. The SCI user was asked to attempt movement from the appearance of the arrow until a blank screen was shown i.e. from 0 to 3 s. The whole EEG assessment was repeated if the user completed the study, consisting of at least 10 BCI-FES sessions total.
- *Range of movement (ROM)* of users’ wrists on both sides was measured before the start of BCI-FES using a digital goniomieter (Biometrics Ltd, UK). The SCI user performed maximum flexion and extension five times and the angle at the maxima was noted. This was repeated if the user completed the entire study including additional sessions.
- *Donning Time* i.e. the time it took to set up the whole system with each use was measured. This was defined as the total of
- placing headset
- achieving good contact of electrodes
- determining the EEG training threshold
- placing FES electrodes
- setting FES stimulation parameters
- testing the threshold and parameters to make sure they are correct
- *Accuracy* of the software in achieving FES activation was monitored. The true positives, false positives, and true negatives were recorded during each session (Eq. 1 and 2). Because of the nature of the algorithm false negatives were not possible i.e. it wasn’t possible for the FES to activate if alpha band power did not remain below threshold for a sufficiently long period of time.
  Equation 1 $$\frac{TP}{TP+FP+TN} \times 100\%$$
  Equation 2 $$\frac{FP}{TP+FP+TN} \times 100\%$$
- *Repeatability of FES parameters:* during each session the following FES parameters were noted: pulse current, pulse width, start time, and duration. These values were then inspected for intersession repeatability.
- *Repeatability of Threshold:* the value of training threshold used during each session was noted and later analysed for intersession repeatability.
- *Number of sessions needed with OT:* total number of training sessions with an OT, before the SCI user and caregiver felt confident in continuing the therapy on their own.
- *Time needed for FES activation:* the time between cue signalling to start AM and actual FES activation, with minimum being 1 s and maximum 15 s.

### Offline EEG analysis

*Multichannel EEG assessment—*artefacts pertaining to eye-blinks or other muscular movements and spasms were removed during visual inspection and using Independent Component Analysis (ICA) in EEGLAB toolbox [37] for Matlab (The MathWorks Inc, USA). Data was epoched and cumulative ERD/ERS [38, 39] scalp maps and spectrographs were produced for AM of all SCI users’ initial assessment for frequencies 3-30 Hz. Baseline period was set as t = −3 s to t = −1.5 s (before warning cue at t = −1 s). Statistical significance was tested using bootstrapping statistics (p = 0.05) and corrected for multiple comparisons.

*Single channel BCI-FES EEG analysis—*time–frequency analysis of EEG recorded during BCI-FES trials using Emotiv EPOC headset was performed to visualise ERD/ERS during trials. Data was bandpass filtered from 2 to 45 Hz, epoched, and epochs with artefacts were manually discarded during visual inspection. Analysis was performed using the Morlet Wavelet transform [40] with Hanning-tapered window applied with a minimum of 3 wavelet cycles per window at lower frequency. Baseline period was set to -2.5 s to -0.5 s i.e. before appearance of execution cue (at t = 0). Statistical significance was tested using bootstrapping statistics (p = 0.05) and corrected for multiple comparisons.

### Statistical methods

The Wilcoxon Signed Rank test was used to test donning times and workload experienced by participants for statistical significance at the 0.05 level. Spearman’s correlation test was applied to the donning times vs workload experienced.

## Results

Results from all quantitative and qualitative outcome measures are presented using the well-established framework for usability which consists of efficiency, effectiveness, and satisfaction [27] as shown in Table 1.

**Table 1:** Categorised outcome measures

Category	Measure	Participants assessed	Assessment frequency
Effectiveness	BCI accuracy	P	ES
	ROM	P	BT, AI
Efficiency	NASA TLX workload	OT, P, C	ES
	FES parameter repeatability	P	ES
	Threshold repeatability	P	ES
	Number of training sessions	OT, P & C	AT
	Donning time	OT, P & C	ES
	Time for each FES activation	P	ES
Satisfaction	QUEST	P & C	AT, AI
	Interviews	P & C	FS, AT, AI
	Focus group	OT	ATT, EoS
	Perceived usefulness Questionnaire	P & C	FS
EEG	EEG assessment	P	BT, AI

Each category and the corresponding outcome measures including the user groups who were assessed using that particular measure—these are P for SCI users only, C for carers only, P&C for SCI users and carers together, and OT for occupational therapists only. Assessment Frequency: ES—every session, FS—first training session, BT—before training sessions with OT, AT—after training sessions with OT, ATT—after OT training (lead by researchers), AI—after at least 10 sessions total (including both training and independent), EoS—at end of study (all P&C sessions completed)

In addition we also show group level ERS/ERD before the therapy to demonstrate how SCI affects the EEG during motor action in early subacute stage. In a previous study [11] we showed that this activity normalises alongside restoration of motor function. In this study, which primarily focuses on the transfer of knowledge, the number of sessions was too small to demonstrate changes in EEG activity.

### EEG assessment

**Fig. 5:**
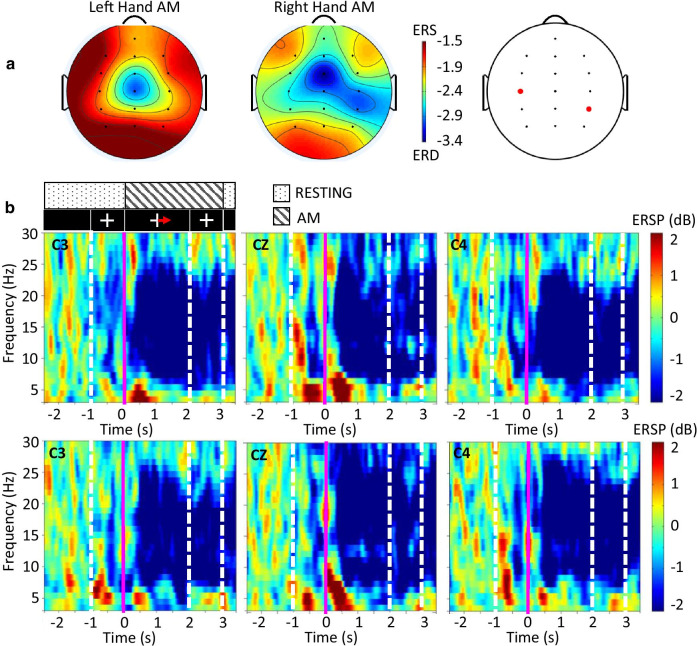
Group EEG characteristics during initial neurological assessment of end users. **a** Group average ERSP of left and right hand attempted movement in 8–24 Hz frequency band. On the electrode map on the far right electrode locations with statistically significant differences between the two conditions are marked with red dots (C3 and CP4). **b** Corresponding group average spectrograms of right and left hand attempted movement. Top row: electrode locations C3, CZ, and C4 during right hand attempted movement; bottom row: electrode locations C3, CZ, and C4 during left hand attempted movement. Above the C3 plot in the top row is a schematic representing what the SCI user was shown on screen (blank black screen, cross only, or cross and arrow to right or left), and what actions were performed during the trial (resting or AM). This sequence applies to all six plots

From the initial EEG assessment the ERD/ERS associated with AM of the hand was plotted for all 8 SCI users. Figure 5 shows the group average ERSP and spectrograms during AM trials.

Collectively, there is no lateralisation during left hand movement, and slight ipsilateral lateralisation during right hand movement as seen in Fig. 5a. These types of EEG patterns are not uncommon in the sub-acute SCI population and may change over the course of recovery using BCI-FES to resemble the contralateral lateralisation seen in the able bodied population [41, 42].

Figure 5b shows ERS/ERD over selected electrodes over the primary motor cortex. Although broad ERD is exhibited after the onset of movement (at 0 s) in alpha and beta bands (approximately 7 to 25 Hz), no lateralisation can be noticed. The ERS (red) is sensory response to visual stimuli and tactile sensation of movement.

**Fig. 6:**
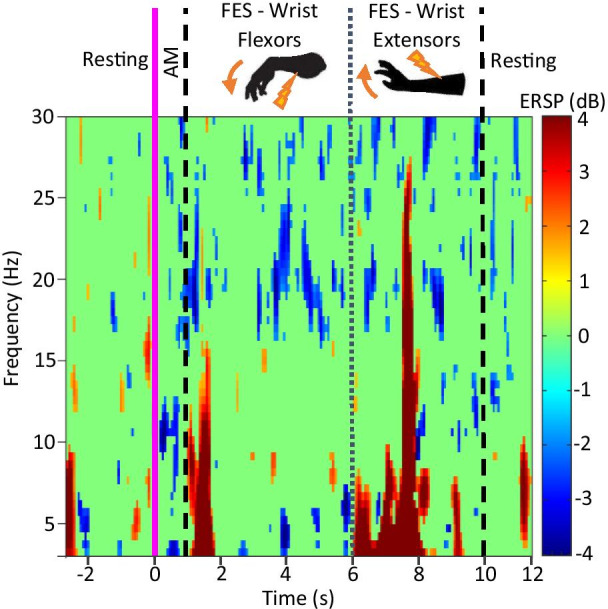
Cumulative time–frequency response of a representative SCI user (P7) during 10 successful BCI-FES trials on the right hand

Furthermore, the EEG recorded by the Emotiv EPOC during the BCI-FES sessions was used to verify activity in the alpha band during the attempted movement phase of the hand therapy along with the lack thereof during other parts of the trials and unsuccessful trials (Fig. 6).

ERD can be observed during AM in the alpha band, but also in the beta and gamma band during AM and subsequent FES. Strong ERS can be observed at the start of stimulation of each FES channel. This can be explained by sensory response related to the onset of wrist flexor FES stimulation from t = 1 s to t = 2 s, and offset of flexor and simultaneous onset of extensor stimulation at t = 6 s [43]. The final component contributing to this large ERS feature is the imprecision of plotting wavelets spectrograms at lower frequencies.

The spectrogram of BCI-FES therapy trials appears quite different than those shown in Fig. 5b for several reasons. Firstly, the user is not only attempting movement but also receiving FES stimulation. Pure AM without stimulation can be seen between t = 0 sand 1 s and the corresponding ERD which lead to FES activation is present in the alpha band. Secondly the EEG response plotted here is that of only a single user; it could not be averaged because of variation in order of stimulation pattern, hand trained, and FES parameters.

### Effectiveness

Due to the small number of therapy sessions it was not possible to fully assess the effectiveness of the therapy and we present only the ROM for three SCI participants who took part in independent therapy sessions. We also present the effectiveness (in this context the accuracy) of using BCI-FES system.

#### The range of movement

Changes in ROM of wrist flexion and extension of three participants who performed a final assessment after their last independent session are as follows:

- P1—left hand (trained) decreased by 7° and right hand (untrained) decreased by 3^o^
- P4—right hand (trained) increased by 23° (left hand untrained, not tested)
- P7—right hand (trained) increased by 9° and left hand (untrained) increased by 16^o^

SCI participants attended conventional therapy in parallel to this study therefore it is not possible to attribute any functional improvement to BCI-FES therapy alone. The changes in ROM may have been caused by the amount of hand stretching each person has done within a day or two before testing.

#### BCI accuracy

**Fig. 7:**
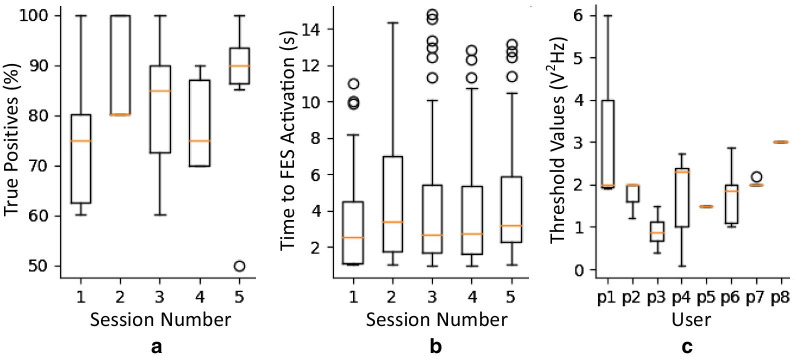
BCI performance and parameters. **a** Percentage of true positives activation out of all attempted trials across all SCI users for first five sessions. **b** Time to activate FES across all SCI users for the first five sessions. The numbers in each box denote the number of data points for each box. **c** Range of alpha power threshold values used by each SCI user during their first five sessions

In Fig. 7a the median percentage of true positives achieved varies between 75 and 90% out of all attempted trials for the first five sessions across all SCI users. The mean individual percentage of false positives for each SCI user ranged from 4.6 to 10.4%

### Efficiency

#### BCI-FES parameters

Time for FES activation and BCI activiation threshold values during first five sessions are presented in Fig. 7. These give an indication of the ability of users to control the BCI program and indicate the learning process.

Figure 7b presents the length of time from the appearance of the cue indicating the start of AM until FES activation occurred. It can be seen that the time needed for each activation varies but the median stays fairly constant across sessions. During the first training session across all SCI users the mean was 3.39 ± 2.55 s, median 2.54 s, min 1.02 s, and max 11 s. During the last training session across all SCI users the mean was 4.10 ± 3.16 s, median 2.76 s, min 1.03 s, max 12.86 s. Figure 7c shows the level of alpha power needed to activate FES stimulation with each trial for the first five sessions of each SCI user.

#### Number of training and independent sessions

All completed sessions by each user pair are shown in Table 2. Four caregivers felt confident enough with the system after 4 training sessions and three after 5 sessions. The median number of training sessions completed by user pairs was 4 (min 1, max 5), and median number of self-managed sessions was 8 (min 4, max 10) excluding pairs who withdrew after training sessions only.

**Table 2:** Number of SCI user and caregiver sessions and completion

User pair	Training sessions	Self-managed sessions	Completion
P1 & C1	4	10	C
P2 & C2	4	4	CT +
P3 & C3	5	–	CT
P4 & C4	5	10	C
P5 & C5	4	–	CT
P6 & C6	5	–	CT
P7 & C7	4	6	CT +
P8 & C8	1	–	W
Total	32	30	–

Training and independent sessions completed by each user pair. In the far right column, CT = completed training, CT+  = completed training and some independent sessions, C = completed training and all independent sessions, W = withdrew before completion of training

#### Donning time

**Fig. 8:**
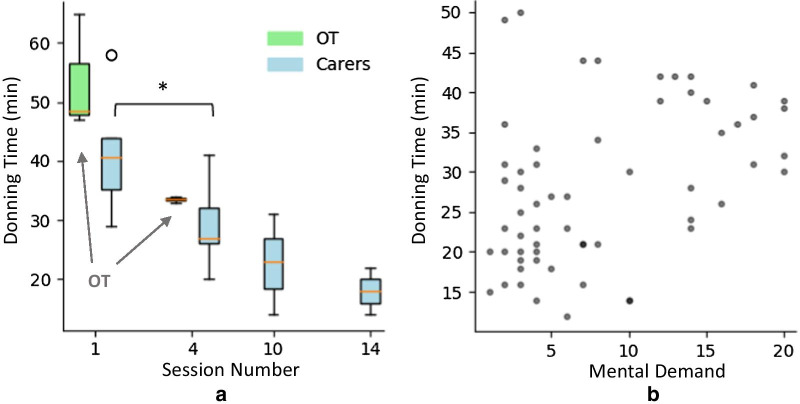
Donning times during sessions. **a** Donning times for all caregivers (donning for SCI user sessions) (blue) for sessions 1 (N = 8), the last training session labelled 4 here (N = 7), however for 3 user pairs the 5th session was the last training, 10th (N = 3) and 14th (N = 2) sessions showing therefore donning times approximately every 4–5 sessions. Donning times achieved by OTs (green) (N = 3) show first and last OT training sessions with researchers. The box for OT’s at last training is hardly visible because there was little variability in the values. **b** Mental demand (min 0, max 20) experienced by caregivers (measured by NASA TLX [34]) at each particular session and the corresponding donning time during that session

The range of donning times for OTs during their training sessions, who practiced the same actions as the caregivers during their training, is shown in Fig. 8a along with donning times for SCI users and caregivers from their training sessions which were instructed by the OTs.

Donning times seen in Fig. 8a show OTs time decreased from median 48 min during the first session of full BCI-FES system setup to 33 min median for the last training session. Carers’ median donning times also decrease with each session, reaching a final value of 18 min at the 14th session. A Wilcoxon signed rank test was performed on the SCI user and caregiver pairs’ donning data for first and last training sessions for 7 pairs who completed training, and the difference was found to be significant (p = 0.018) at the 0.05 significance level.

Figure 8b shows that the mental demand experienced by carers influences the resulting donning time for a particular session (Spearman’s correlation ρ = 0.479, p value = 0.0001). Since donning time is an important aspect of usability, this suggests improvements to lower the mental demand of the carers would positively influence the overall usability of the system.

#### Workload

**Fig. 9:**
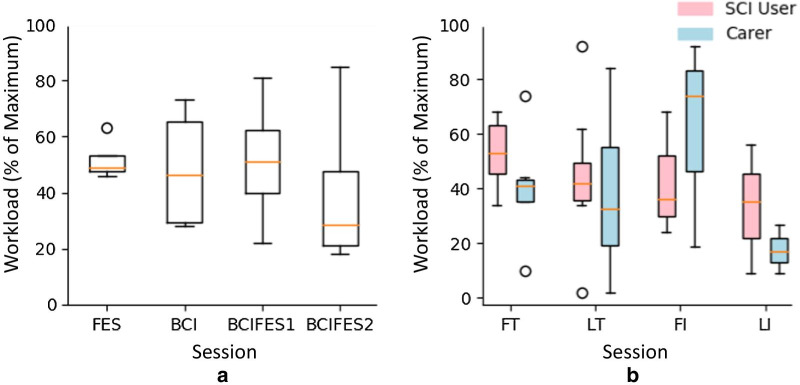
Workload experienced during sessions. **a** OT workload during each of their training sessions - each box consists of 4 data points. **b** Overall workload for the first and last training and independent sessions for all carers and SCI users. The workloads are presented as % of maximum possible score (120) for both (**a**) and (**b**)

Figure 9 shows the reported workloads during the sessions as measured using the NASA TLX questionnaire [34]. The OTs had four training sessions which have been separated by content, and similar values were reported for total workload during the first three sessions covering FES only, BCI only, and full BCI-FES setup with medians of 41, 40, and 43 percent of maximum respectively (Fig. 9a). For the second and final full BCI-FES setup session the median was considerably lower at 24 percent of maximum, i.e. workload has nearly halved from session 3 to session 4.

Figure 9b shows that the workload for SCI users generally stays more constant than carers and medians decrease with each session plotted, though due to low number of participants in independent sessions, the exact trend could not be established. Interestingly, for carers the workload peaks at the first independent session and is clearly the lowest for the last independent session, again bearing in mind the reduced number of participants. No significant difference was found between the first and last training session workloads for neither SCI users nor carers (Wilcoxon signed rank test, p = 0.46 and p = 0.84 respectively). Differences between other sessions could not be tested due to the limited number of data points.

#### Intersession repeatability of FES parameters

For a therapy delivered by non-professionals, it is important that system parameters are as stable as possible. The FES parameters used by each SCI and caregiver pair were initially established by the OT during the training sessions. It was found that these remained unchanged for 70% of training sessions and 84% of independent sessions. The changes that were made by the participants most often was altering the stimulation current intensity. Across all users, the current settings were altered by a median value of 4 mA (min 0, max 8).

### Satisfaction

#### QUEST

The results of the QUEST questionnaire SCI users and carers completed after the last training session with OT showed that the most important aspects to users were ‘Easy to Use’ and ‘Effective’. Across users, the BCI-FES system in the present study was rated as 3.43 ± 0.90 for ‘Easy to Use’ and 3.29 ± 0.70 for ‘Effective’ (both out of 5 max). When rating satisfaction with the BCI-FES used in the present study, the users were most satisfied with ‘Safe and Secure’ at 4.43 ± 0.9, ‘Weight’ at 4.43 ± 0.9, and ‘Professional Services’ at 4.43 ± 0.73. The average satisfaction with the BCI-FES system was 3.68 ± 0.81 out of 5, and with services provided was 4.14 ± 0.82 out of 5.

#### Interviews and perceived usefulness questionnaire

**Fig. 10:**
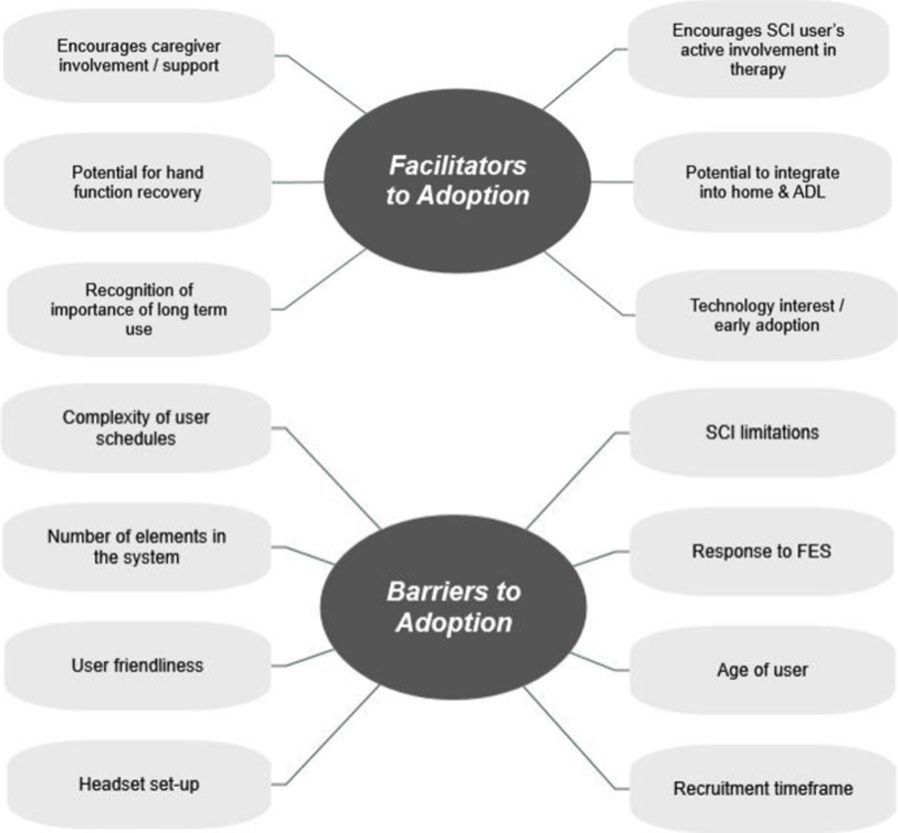
Barriers and facilitators to BCI adoption. Categories derived from interviews with SCI users and caregivers and focus groups with OTs

The results presented in this section combine both qualitative data, in the form of quotations from participants, as well as quantitative data, in the form of ratings (scale 1–10) contained in the Perceived Usefulness Questionnaire. These results are presented as either barriers or facilitators to BCI-FES adoption by the end users and their caregivers and are summarised in Fig. 10.

#### Facilitators

OTs and caregivers both identified the caregivers’ willingness to help and be involved in the SCI users’ recovery as a motivating factor to using the BCI-FES:

“…they felt like they were able to help and do something for their loved ones, whereas maybe not being so involved with their rehab they feel like they are a bit helpless.”—OT3.

“I like to be involved, that’s what I like about it.”—C7.

Both SCI users and caregivers were encouraged by the sight of the user’s normally limited hand moving due to the FES:

“I like the fact that I can see his hand working. It’s kind of hard to believe these things until you actually see it working.”—C7.

For SCI users and caregivers another motivator was the potential for functional recovery of hand movement. Some users and caregivers who completed additional sessions attributed a degree of recovery to the BCI-FES use:

“I can see a lot more flexibility in the hand than when we started and I believe what we have done has contributed to that and is of benefit.”—C4.

Conversely, when OTs were asked if they saw and improvement in SCI users hand function due to BCI-FES they replied:

“No, I couldn’t say. No”—OT1, OT3.

Later, OTs also acknowledged the difficulty of distinguishing the potential source of any recovery because of natural recovery also taking place.

“…no matter what, you can’t really tell if the functional gains are because of the intervention.”—OT2.

Nevertheless, SCI users and carers gave an average rating of 7.9/10 (1 = not at all, 10 = very much convinced) for the perceived usefulness of BCI-FES in improving their muscle strength and hand function, showing the general belief in the potential benefit of the therapy. In addition, OTs also confirmed this to be a motivating factor for people with SCI:

“I think most of our patients are quite motivated, if they think it is going to help them.”—OT1.

One aspect of the BCI which OTs saw value in was active involvement of SCI users in their therapy.

“I think the fact that the patient is a bit more active in it, thinking about it and initiating that movement encourages them to be proactive. Often patients can be passive. They often just like to have their hands going up and down and see their hands moving but at least with the BCI element, they are thinking about it and trying to engage.”—OT1.

OTs also raised the importance of any at home therapy being integrated with the person’s home environment and ADL:

“It’s really just about encouraging them to use their hands as much as possible, so in the shower getting washed, making things in the kitchen, going out and about with their family friends whatever their lifestyle was beforehand.”—OT1.

Finally, when asked about their readiness to try new technology, 3 out of 7 SCI users and 1 out of 7 caregivers self-reported being early adopters, making them more likely to use the BCI-FES in general. Attitude towards new technology was rated as 7.6/10 on average across all SCI users and caregivers (Perceived Usefulness Questionnaire; 1 = extreme avoidance, 10 = extreme excitement). OTs were of the opinion that caregivers with this inherent interest were more likely to continue BCI use at home.

“I think for the ones who already had a technology background…because I think they are already interested in it, then they are more likely to use it. But I think for the ones who didn’t really get it and were just doing it for their loved ones, to be helpful, I couldn’t really see them going out of their way to look for other gadgets to do it [continue therapy].”—OT3.

#### Barriers

With regards to barriers to adoption of the BCI-FES system, there were some comments from OTs and caregivers regarding a lack of time to use the system when at home. This was believed to be caused by the sheer number of things which happen when a person is discharged from the hospital as well as the busy schedules of caregivers:

“I just think, to even think about taking it home, there would be so much going on. It’s going to be hectic, especially at first when he [P6] comes home.”—C6.

“I think when you have the added factor of having a carer needing to set you up, it makes it even less likely that it would be continued at home apart from a small percentage of focused driven people.”—OT2.

Another barrier identified by all user groups was the time consuming nature of the BCI-FES therapy, complexity, “*just the number of elements*” (OT2), and low user friendliness:

“Length of time it takes to set-up and issues with trying to find the points on the head I think would make it more of a thought for them having a session.”—OT1.

‘The real problem that I would have is the software. To remember one or two things, or to save something and then you can’t move on. I would need to have it written down step by step.”—P2.

Other difficulties discouraging for independent use related to the EEG headset donning:

“The hardest bit for me is positioning the headset on his head and getting the green dots [good electrode contact].”—C5.

“… [P1] has so much hair, it’s just trying to get a good connection.”—C1.

Despite these comments, participants generally reported that the system was fairly easy to use given enough practice: “Once you have a good few weeks, it just takes a bit of practice. I feel more confident now.” (C7). In the Perceived Usefulness Questionnaire participants rated ‘easy to use’ as 6.7/10 on average (1 = very difficult, 10 = very easy), showing that with practice users overcame these obstacles.

The health condition, response to FES, and potential for recovery related to age of the participants was also identified by OTs as problematic for BCI-FES use:

“We seem to be having a lot of patients that are not really responding as well to FES and older, less potential in terms of their rehab as well so it was quite difficult.”—OT3.

Furthermore, OTs identified age of the user had an influence on their experience, because of the degree “of their [P’s & C’s] technological abilities” (OT1), eyesight, and other conditions:

“The laptop, the screen, everything has to be bigger, that is the main thing. (…) It’s quite difficult and I think as well for the older population, if their hands are a bit shaky (…) having to touch the screen would be quite difficult.”—OT3.

Other barriers identified pertained to recruitment of participants during the sub-acute stage after injury as OTs mentioned the limited timeframe for potential recruitment:

“I think it is a narrow window of opportunity for the recruitment because at some points it’s too early and they are emotionally not ready or their sitting time is limited or they have other things going on that by the time they are ready it might be getting close to discharge and just it’s that sort of narrow window.”—OT2.

When participants were asked how/where they would look for rehabilitation technology the majority suggested looking on the internet for information as well as speaking to occupational therapists for advice:

“Probably Google and also therapists”—P7.

However, OTs were cautious to recommend therapeutic technology since each person had individual needs and recovery goals and no one device could address them.

“I think you also need to think about why is it you are recommending it? Is it just to do an activity and is there any outcome to doing that activity?”—OT3.

Another reason stated was to lead the people with SCI to accept their new state and its limitations.

“And it’s trying to not mislead them that they will need rehab forever, because some of them like to think they might (…) so giving them that closure as well is really important.”—OT4.

## Discussion

In the present study we have shown the feasibility of the transfer of knowledge from researchers to therapists and onwards to caregivers and users with SCI which, to our knowledge, is the first such instance in published literature. At the end of the study OTs reported confidence in using the system and teaching it to SCI users and caregivers. With enough practice, appropriate learning aids and instructions, and lasting remote support, therapists could continue to teach people in need of the therapy and their caregivers, and could even pass on the knowledge to other therapists.

Those possibly most in need of such an accessible home based therapy are people living in rural and remote areas who do not have physiotherapy clinics nearby. Therefore, in order for any home based therapy to be a truly accessible and sustainable solution, it is essential to organise training locally and provide local and remote support. Hence, why the researcher-therapist-caregiver transfer of knowledge is essential in this context.

Considering our second objective, we have demonstrated satisfactory usability of BCI-FES as a self-managed tool for hand therapy among people with SCI and their caregivers. Other BCI studies which have involved caregivers have not assessed their performance and experiences to this degree [15, 44]. The detailed evaluation of each of the three usability constructs follows.

### Efficiency

All user pairs felt confident that the four or five training sessions with an OT were sufficient to continue using the system independently, however results suggest users are still learning at this point. It appears the users learn first by instruction from the OT and documents and at the end of training they feel comfortable to continue learning, but now from one’s own mistakes. Hence further supervision is required and users should always have access to contact an ‘on call’ expert while using such a system at home [44, 45].

The EEG device and setup has been highlighted as an area for improvement in numerous BCI usability studies [17, 28, 46]–48. Although the Emotiv EPOC device used here presents an improvement upon the traditional cap and gel solution, the headset still proved to be a difficulty. However, with practice all caregivers managed to achieve correct placement and users were able to control the neurofeedback. This is a significant finding since people with SCI living at home, have limited time with a professional caregiver or relative, and during the time they have ADL take priority. Hence, the quicker and easier the setup involving the carer, the more feasible the device for daily use for the end user.

The exact EEG alpha band power threshold values appear to be reused or remain within a small range leading to good inter-session reliability for each user. Similarly, FES parameters stayed constant with the same value being reused for the majority of all sessions for all SCI users. The stability of both of these parameters is promising in the context of self-managed use in a home setting.

The medians of workloads reported by users during the study were low to moderate, confirming the operation of the entire BCI-FES system was not too difficult for the users to learn.

### Effectiveness

Even though some SCI users perceived an improvement in hand function, it cannot be concluded to be a result of the BCI-FES therapy because of several factors. Firstly natural recovery occurs after injury and each SCI user was actively attending physiotherapy and occupational therapy sessions during the course of the study. Secondly, the BCI-FES intervention was too short to expect any meaningful results as the minimum number of sessions would be approximately 10 long sessions or 20 brief ones [9, 11, 49].

Nevertheless, it has been shown that perceived usefulness of assistive or rehabilitative technology is one of the primary factors in predicting actual use of the device [50, 51]. In the present study, users positively evaluated the perceived effectiveness in strengthening muscles and improving hand function.

The accuracy of the BCI has been shown to be comparable to other BCI studies [18, 44, 46], indicating that the simple threshold time switch is an effective classifier in this case. This is encouraging as it has been shown that those who perform well with BCI are more interested in using one [15].

### Satisfaction

The results from the QUEST questionnaire overlapped with other BCI studies which implemented the measure, which also highlighted ease of use and effectiveness as the top factors in determining satisfaction with an assistive device. The cumulative satisfaction scores with the device were in a similar range to other studies with most users being generally satisfied [17, 18, 28, 45, 46, 52].

### Facilitators and barriers to BCI adoption

Finally, in relation to our third objective, the factor which arguably encouraged the participants to use the BCI-FES system the most was the potential benefit in recovery of hand function.

Caregivers seemed to be motivated by two thoughts: one being the potential for improvement in function of their loved one, while the other was the thought of being able to have an active role in their recovery. Therapists however did not think this would be enough motivation for further continued BCI-FES use at home.

Another factor facilitating the adoption seems to be younger age of the participants and high computer literacy, as has been shown in other studies investigating technology adoption [53]. Younger caregivers with an interest and experience with technology performed much better than older caregivers, however with time currently young and tech savvy will become older and this may no longer be the trend. The only obstacle then will be the cognitive ability.

The OTs involved were motivated by the aspect of the necessity of active engagement of the people with SCI in the therapy. They saw it as beneficial for the person’s recovery as they regularly encourage people to take an active role but many remain passive while receiving FES in conventional therapy.

Finally, OTs also identified a potential factor influencing long-term use of the BCI-FES in a home setting as therapy its ability to produce complex movement patterns for ADL, which form a core of conventional community therapy for people with SCI and stroke [54, 55]. Other studies that have incorporated hand therapy into ADL have seen better adherence by the participants than those which have required users to specifically dedicate a significant amount of time for therapy [52].

Among barriers to BCI adoption identified, the need for the system hardware and software to be user friendly and easy to use echoes other BCI studies with end users [17, 47, 48]. Despite the Emotiv EPOC EEG device used here marking a significant improvement in ease of use upon the traditional cap and gel solution used in other studies [48], it still proved to be difficult for the users. Other user friendliness aspects pertaining to software should be addressed with the relevant improvements to make the system simpler and more intuitive.

A common obstacle for rehabilitation and assistive technology use at home is the users’ general health, as mentioned by OTs in the present study. In studies using BCI for communication with locked-in users deteriorating health was also identified as a reason for abandonment [44]. Ill health was the cause of decreased use or withdrawal in studies using a soft robotic glove [52] and BCI for neurofeedback [45] at home by people with SCI.

Recruitment issues mentioned by OTs may not be applicable for self-managed therapies in people with chronic SCI at home as these are specific to the sub-acute phase after injury. Similarly, distance travelled by caregivers to take part in the present study made participation stressful and demanding for those who lived further away from the hospital, while if the therapy takes place at home distance would not be a consideration.

Finally, a discourse between the end users’ interest in trying new rehabilitation technology and the therapists’ reluctance to recommend it has been identified previously [21]. OTs in the present study mentioned the road to acceptance of the limited abilities after SCI, similar to stroke as people after a sudden traumatic experience go through a period of adjustment to injury, therapists and psychologists provide support and guidance through this time [56].

### Limitations

Studies involving people with SCI tend to have relatively few participants because of the low prevalence of such injuries compared to stroke for instance. Here seven participant pairs completed the BCI-FES training sessions, which is comparable with other BCI usability studies [17, 48], though a total of 18 individuals (7 participants with SCI, 7 caregivers and 4 OTs) learned to use the system. While a larger number of participants would have made some results more conclusive, these numbers are within the recommended range for usability studies in general, as some suggest only 5 participants is enough [27].

Only three user-caregiver pairs completed enough self-managed sessions to perform a final assessment, therefore effectiveness could not be thoroughly investigated. Furthermore, the lack of a control group prevented the assessment of the degree of improvement due to BCI-FES versus conventional therapy and natural recovery.

In order to assess usability of the system for self-managed home use, the system should be tested in the setting where it will be used, i.e. at home. However, the present study took place in a research space within a hospital therefore various aspects inevitably differed. The amount of environmental noise affecting the EEG device and focus of the user may have been less than that encountered at home, which could lead to potential issues in BCI performance in such a setting. The caregivers also had to travel, some a significant distance, and this likely impacted their experiences and satisfaction in using the system. Furthermore, the fact that both the training and self-managed sessions were supervised by OTs and researchers may have had an impact on the pressure caregivers and SCI users felt to perform, hence raising the level of stress as opposed to a one-to-one session with an OT.

Because of the number of participants required to carry out a BCI-FES session (SCI user, caregiver, OT, researcher), all of whom had busy schedules, often finding a suitable time for all involved proved to be a challenge. Hence some user pairs’ sessions were sparse leading to more challenges in donning and BCI use simply because of the amount of time separating two consecutive sessions.

## Conclusion

This study demonstrates the feasibility of the handover of BCI technology from the clinical environment to the users’ day to day life. We have shown effective transfer of knowledge from researchers to OTs to users and caregivers, the ability of caregivers to learn and administer BCI-FES therapy, and satisfactory BCI performance. User feedback has reaffirmed the importance of the inclusion of all interest groups in the community healthcare technology design process and provided key aspects for future directions. There must be undeniable evidence of its effectiveness and clinical benefit to people after SCI in order for a solution such as the BCI-FES system to be widely accepted. Randomised studies are needed to investigate the extent of recovery prompted by the system. Finally, development of accessible rehabilitation technology in general should place particular focus on user friendliness and minimal dedicated time for setup and use, ideally incorporating device use into ADL.

## Data Availability

Raw EEG data are available from the authors on reasonable request.

## Abbreviations

EEG: Electroencephalography
BCI: Brain computer interface
FES: Functional electrical stimulation
SCI: Spinal cord injury
OT: Occupational therapist(s)
AM: Attempted movement
QUEST: Quebec User Evaluation of Satisfaction with Assistive Technology
ADL: Activities of daily living
UCD: User centred design
AIS: ASIA (American Spinal Injury Association) Impairment Scale
ERS/ERD: Event related synchronisation/desynchronization
NASA TLX: NASA Task Load Index
ROM: Range of movement
TP: True positive
FP: False positive
TN: True negative
ICA: Independent component analysis
GUI: Graphical user interface
MMT: Manual muscle test
